# A Catalogue of the Medicinal Plants, Indigenous and Exotic, Growing in the State of New York, with a Brief Account of Their Composition and Medical Properties

**Published:** 1848-02

**Authors:** 


*A Catalogue of the Medicinal Plants, Indigenous and Exotic, growing in the State of New York, with a brief account of their composition and medical properties.* By Charles A. Lee, M. D., Professor of Materia Medica in Geneva Medical College and the University of Buffalo. *Pamphlet of* 64 *pages.* New York: 1848.

This is a valuable *brochure*, for which we are sure the author will receive the thanks of his professional brethren, and especially those of his own state. In his arrangement, the author has followed the natural system, "as the only one which can serve as a useful guide to the medical man in pursuing the study of this interesting science;" he has, however, "omitted the botanical description of the different orders, genera, and species," to avoid extending the catalogue beyond the limits assigned to it. When will some industrious cultivator do a like service for Pennsylvania? The valuable works of Barton and Darlington would afford essential aid for such an undertaking.

